# Tape-Releasing Suture with “Long Loop” on Mid-Urethral Sling: A Novel Procedure for Management of Iatrogenic Urethral Obstruction

**DOI:** 10.3390/jcm12123938

**Published:** 2023-06-09

**Authors:** Cheng-Yu Long, Chieh-Yu Chang, Yi-Yin Liu, Zi-Xi Loo, Chang-Lin Yeh, Ming-Ping Wu, Kun-Ling Lin, Feng-Hsiang Tang

**Affiliations:** 1 Department of Obstetrics and Gynecology, Kaohsiung Municipal Siao-Gang Hospital, Kaohsiung Medical University, Kaohsiung 80708, Taiwan; 2 Department of Obstetrics and Gynecology, Kaohsiung Medical University Hospital, Kaohsiung Medical University, Kaohsiung 80708, Taiwan960233@kmuh.org.tw (K.-L.L.); 3 Department of Obstetrics and Gynecology, Kaohsiung Municipal Ta-Tung Hospital, Kaohsiung Medical University, Kaohsiung 80145, Taiwan; 4 Department of Obstetrics and Gynecology, Chi Mei Foundation Hospital, Tainan 71004, Taiwan; 5 Department of Obstetrics and Gynecology, College of Medicine, Kaohsiung Medical University, Kaohsiung 80708, Taiwan

**Keywords:** urethral obstruction, tape-releasing suture, mid-urethral sling

## Abstract

Background: To report our experiences of a tape-releasing suture with “long-loop” in women with iatrogenic urethral obstruction following the mid-urethral sling procedure. Methods: A total of 149 women underwent a tape-releasing suture with “Long Loop” during the operation. Post-void residual volume was evaluated after Foley removal. Lower urinary tract symptoms and urodynamic studies were assessed before and six months postoperatively. Results: Nine women out of 149 who underwent mid-urethral sling surgery were found to have iatrogenic urethral obstruction post-operatively based on their urinary symptoms and ultrasound findings. There was no apparent difference between tested groups in mid-urethral sling products and concomitant procedures. 77.8% had successful releases after the first Long-loop manipulation procedure, and 22.2% required two or more releases. However, the SUI cure rate is similar in groups receiving the Long-loop manipulation or not (88.9% and 87.1%, respectively). Conclusions: We are convinced of the practicability and efficacy of the tape-releasing suture “Long-loop.” We adopted subjective and objective means to evaluate both groups before and after a six-month follow-up. The Long-loop manipulation procedure can successfully resolve the iatrogenic urethral obstruction without compromising the effectiveness of mid-urethral sling for the treatment of SUI.

## 1. Introduction

Stress urinary incontinence (SUI) affects women with high prevalence and profoundly impacts their quality of life. Though the pathophysiology and mechanism are well-known in the current medical field, the incidence can be underestimated since some women find it too embarrassing to talk about or fearful of receiving surgery [1]. Various conservative SUI treatments such as behavioral and lifestyle modifications, Kegels’ exercises, urethral bulking agents, and vaginal lasers had been offered but with limited effectiveness and high recurrent rates. And so far, no pharmacological agents have demonstrated sufficient effectiveness in treating SUI [2]. In contrast, the surgical treatment offered the highest success rate among all other conservative treatments within a rapid time besides the operational risks. However, mid-urethral sling procedures are the standard surgery among all surgical treatments for their high effectiveness and minimal invasion [3].

In 1995, Ulmsten and Petros introduced a minimally invasive surgery for female stress urinary incontinence (SUI), namely the tension-free vaginal tape (TVT) procedure [4], with reasonable subjective and objective cure rates (85–90%) after more than 17 years of follow up [5]. Based on the integral theory, continence can be achieved by placing a vaginal tape underneath the mid-urethra without tension to reinforce the weakened pubourethral ligament [6]. In an attempt to reduce the morbidity of bladder injury, an alternative approach with the transobturator route of the Prolene tape (TVT-O) was developed by De Leval in 2003 [7]. Theoretically, the trans-obturator tape lies in the mid-urethra at a less acute angle than the TVT, and it is less likely to obstruct urine flow during voiding. However, women with iatrogenic urethral obstruction are still reported clinically [8,9].

The single-incision sling system (SIS) shares a mechanism similar to TVT-O, lying below the mid-urethra but without penetrating the bilateral obturator externus muscle, which can be a promising treatment option for female SUI [10]. The advantages of The SIS are comparable success rates with fewer complications such as wound pain, hemorrhage, and nerve injury, and less foreign material stays in the human body [11]. Although SIS is potential in current treatment options, postoperative voiding dysfunction following the SIS procedure is still a possible complication. The postoperative voiding dysfunction can be frustrating to the surgeons and painful to the patient, eventually deteriorating the trust between doctors and patients.

Reported risk factors for voiding difficulties after anti-incontinence surgery in published studies, including elderly (age over 65), the type of surgical procedure, concomitant surgical procedures, abnormal preoperative voiding studies, and postoperative infection [12]. The iatrogenic obstruction can mostly be managed with conservative treatments, including timed voiding and self-clean intermittent catheterizations. If failed, more invasive treatment options include urethral dilation, urethrolysis, and excision of the vaginal tape [13,14,15,16].

Urethral dilation can be performed with a Hegar dilator in the outpatient setting; however, the success rate is not as satisfying but likely to have recurrent stenosis, bleeding, and scarring [17]. Moreover, the patient may be terrified and painful during the dilation procedure. Urethrolysis, a surgical approach, is often reserved until the last step, and irritative bladder syndromes may persist afterward. Excision of the vaginal tape is the quickest and most efficient way to resolve postoperative iatrogenic obstruction. However, these invasive surgical approaches might compromise the effectiveness of the mid-urethral sling and lead to recurrent SUI. Vaginal excision of the sling is a quicker and more effective procedure than urethrolysis [13,14,15,16]. However, the destruction of the sling and vaginal epithelium remains a hesitation for the operators.

In the present study, we report a novel procedure using a tape-releasing suture with “Long Loop” on the mid-urethral sling for nine patients with voiding dysfunction and urethral obstruction following the anti-incontinence mid-urethral tape procedures in order to reduce the tissue damage for both sling, vagina, and urethra.

## 2. Materials and Methods

Between March 2019 and November 2020, we collected one hundred and sixty-eight women who underwent the mid-urethral tape procedures for urodynamic stress incontinence (USI) or mixed incontinence with predominant USI in this study. Randomization was performed at the moment of inclusion by a nurse blinded to the patients’ histories. However, we had to exclude the following:

1. No tape-releasing suture “Long-loop.” attachment
2. Incomplete medical records for pre-operative evaluations
3. Incomplete medical records for scheduled follow-ups

Ultimately, this study was conducted based on 149 available subjects, and they were divided into two groups- one with iatrogenic urethral obstruction occurred (9 subjects) and one without obstruction (140 subjects). The comparison of the two groups consisted of clinical evaluations, including a detailed history before and six months after surgery, urinary analysis, pelvic examination using the POP-Q system [18], urodynamic study (UDS), and personal interview to identify urinary and sexual symptoms with the Overactive Bladder Symptom Score (OABSS) [19] and the short forms of the Urogenital Distress Inventory (UDI-6), the Incontinence Impact Questionnaire (IIQ-7) [20], and the “International Consultation on Incontinence Questionnaire-Short Form” (ICIQ-SF) [21]. The standardized questionnaire assessed urinary symptoms using the 2002 ICS definitions [22].

These mid-urethral sling kits included TVT-O (Gynecare TVT-Obturator System, Ethicon, Inc., Somerville, NJ, USA), Solyx (Boston Scientific, Marlborough, MA, USA), and Ophira (Promedon, Cordoba, Argentina). Concomitant transvaginal mesh (TVM) surgeries were performed as needed.

Urodynamic studies, including non-instrumented uroflowmetry, filling and voiding cystometry, and urethral pressure profilometry, were performed according to the recommendations of the International Continence Society with a 6-channel urodynamic monitor (MMS; UD2000, Enschede, Netherlands). Any uninhibited detrusor contraction during filling cystometry was deemed positive for detrusor overactivity (DO).

A Foley catheter would be placed in the urethra in the operation room after the mid-urethral sling procedure to let the patient rest and avoid wound pain. According to the procedure, the Foley catheter would be removed 24 to 48 h post-operatively. After removing the foley, we educated the patient to have adequate water intake, and after a natural urination attempt, we performed a bladder ultrasound. The diagnosis of urethral obstruction following the mid-urethral sling procedure was based on the estimated post-void residual urine (PVR) tests via bladder ultrasound. If the estimated PVR test ≥ 100 mL for two consecutive measurements, then iatrogenic urethral obstruction was diagnosed. Less than 50 mL of estimated residual urine for two consecutive measurements was considered a standard estimated PVR test. A few more tries and single catheter drainage will be considered for those whose post-void residual urine test is 50 to 100 mL.

One qualified urogynecology specialist from Kaohsiung Medical University Hospital contributed all tape-releasing sutures with the Long-loop performed in this study. All procedures of placing the mid-urethral sling follow the standard instructions of each product with an addition of a tape-releasing suture (Long-loop) attachment to the mid-urethral sling. The Long-loop is prepared by appending the 2-0 PDS vertically through the middle half of the mid-urethral sling in the midline. A loose suture is then made with a square knot surrounding two human fingers (index and middle finger). We create a vertical vaginal incision below the mid-urethral and place the mid-urethral sling in a satisfying position. The free end of the Long-loop is exteriorized through the vaginal epithelium incision, which was then closed with simple interrupted sutures by 2-0 Vicryl.

**Figure 1:**
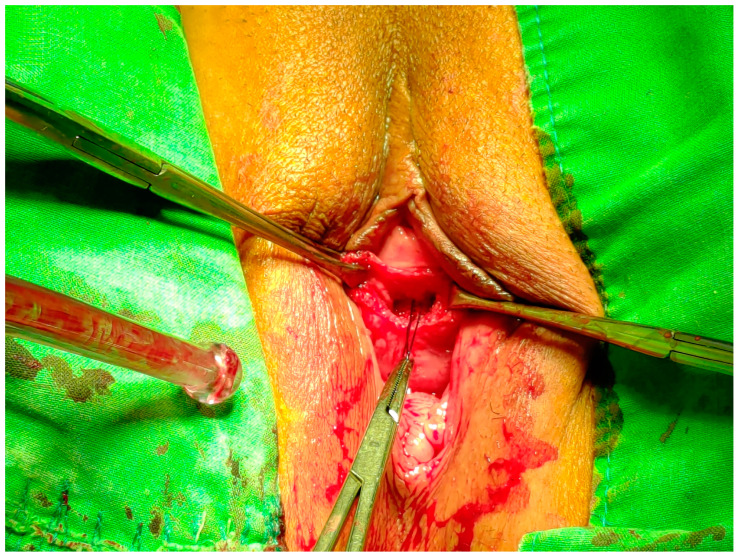
Schematic diagram of surgery.

As releasing the mid-urethral is needed, the patient is placed in a dorsal lithotomy position in an outpatient setting. The Long-loop manipulation procedure is to gently pull the exposed Long-loop downward using a Kelly hemostatic forceps holding the knots. The long-loop manipulation procedure resolves iatrogenic urethral obstruction by traction of the deformable sling in the midline to loosen and lengthen the sling before fibrosis begins. Bilateral anchoring tips remain original without moving or cutting through any tissue. Therefore, the procedure can be performed simply in an outpatient, painless setting. Afterward, the Long-loop manipulation procedure is deemed successful if the woman has estimated residual urine of less than 50 mL. The operation diagram showed in Figure 1.

The Ethics Committee of Kaohsiung University and Teaching Hospital approved the study protocol. Informed consent was obtained from all participants before surgeries. The student’s *t*-test, Fisher’s exact test, and Chi-square test were employed to compare clinical backgrounds between the group with the tape-releasing procedure and the group without the tape-releasing procedure. The student’s *t*-test, Fisher’s exact test, Chi-square test, and paired *t*-test were employed to compare the differences between urinary symptoms and urodynamic parameters pre- and post-operatively. All analysis was performed in a blinded manner. A difference of *p* < 0.05 was considered statistically significant.

## 3. Results

The demographic and perioperative data of all participants were summarized in Table 1. The mean age of our study groups was 59.4–64.6 years old, with a history of mean parity of 2.8–3.1 times and a mean body mass index of 25.0–25.3. Follow-up months were 21.4–24.8. There was no apparent difference between tested groups in mid-urethral sling products and concomitant procedures (Table 1).

**Figure 2:**
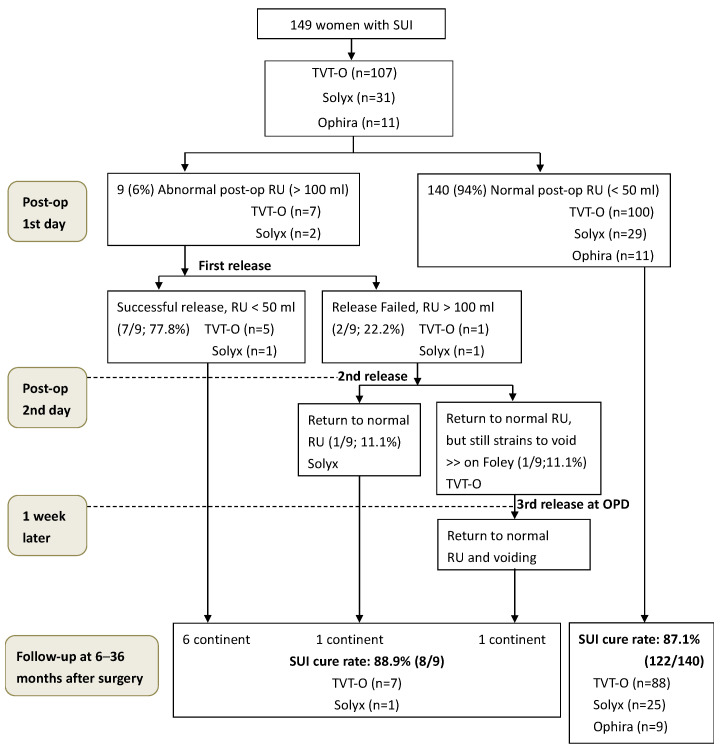
Flow chart outcomes after women had mid-urethral sling surgery. SUI = stress urinary incontinence; Post-op = postoperative; RU = residual urine; OPD = outpatient department.

Post-operative day one, nine patients (6%) had abnormal post-operative estimated residual urine (>100 mL), while one-hundred and forty patients (94%) had a satisfied estimated residual urine (<50 mL), shown in Figure 2. Seven received TVT-O as mid-urethral sling among the nine patients, and two received Solyx. After the first release, seven patients successfully urinated smoothly with the standard PVR test. Two patients required a second release on post-operative day two, and one of them had the satisfied PVR test afterward. Due to difficult voiding, the Foley catheter was placed on the patient who failed the second release (receiving a TVT-O as a mid-urethral sling). The third release was performed at the outpatient department a week later successfully. The patient returned to normal estimated residual urine and easy voiding eventually. In the present study, 77.8% had successful releases after the first Long-loop manipulation procedure, and 22.2% required two or more releases.

At the follow-up exams at six months post-operation, eight out of nine patients who underwent one or more Long-loop manipulation procedures remained continent. The one who became incontinent at six months follow-up received Solyx as a mid-urethral sling in the first place and had one Long-loop manipulation procedure. The SUI cure rate was 88.9%. As for the 140 patients who did not receive any tape-releasing procedure, the SUI cure rate was 87.1% (Figure 2).

Nine patients with iatrogenic urethral obstruction suffered from straining to void and estimated postvoid residual urine >100 mL for two consecutive times. However, none have remained symptoms after the Long-loop manipulation procedure. Eight had frequency or urgency after the surgery, while only four had before the surgery. Only one remained to have symptoms after the Long-loop manipulation procedure (Table 2).

Among the hundred and forty patients without a Long-loop manipulation procedure, the symptom of OAB, questionnaires such as OABSS, UDI-6, IIQ-7, ICIQ-SF, and pad test all showed significant improvement after the operation. Similarly, the group that received the Long-loop manipulation procedure, except for the symptom of OAB, all showed significant differences after the operation (Table 3).

Urodynamic changes compared between both groups before and six months after the operation (Table 4), detrusor overactivity (DO), and first desire (FS) were significantly improved after the operation within the group without Long-loop manipulation procedure. Also, both groups had insignificant differences in maximum cystometric capacity (MCC) and detrusor pressure at peak flow (Pdet) before and six months after the operation. Intuitively, a mid-urethral sling aims to support the sphincter and ligament, which should have little impact on the detrusor function.

There was no difference in maximum flow rate before and six months after operation in both groups. The residual urine before and six months after in both groups showed no significant difference, all under 50 mL. These results could convince us of the capability of the Long-loop. At last, there was no significant difference in functional urethral length and maximum urethral closure pressure six months after the operation in both groups. The maximum urethral closure pressure in Table 4 suggested no intrinsic sphincter deficiency diagnosed from the urodynamic criteria.

## 4. Discussion

Mid-urethral sling placement is highly dependent on the surgeon’s judgment and technique regarding the urodynamic exam and general evaluation. Logically, a proper sling tension provides adequate support to the mid-urethral. Surgical failure usually follows inadequate tension, while iatrogenic urethral obstruction follows the overpowering tension. Nonetheless, it is very challenging to determine the ideal distance and tension between the tape and urethra for a new mid-urethral sling performer. Some authors have suggested inserting a Kelly clamp between the urethra and tape to prevent voiding dysfunction [23].

The incidence of iatrogenic urethral obstruction caused by over-tensioned mid-urethral sling was variable among current studies (approximately 1–10% concluded by the American Urologic Association) [24], and the incidence of iatrogenic urethral obstruction in our study is comparable (6%). However, it is believed that the incidence is under-reported. Surgeons would try to avoid surgical failure; meanwhile, patients often prefer staying dry to wet [25]. The risk factors mentioned in prior studies had no evident impact on our study, which could be related to our relatively small sample sizes. Further investigation with larger sample sizes and longer follow-ups is necessary.

The risk factors mentioned in prior studies had no evident impact on our study, which could be related to our relatively small sample sizes. Further investigation with larger sample sizes and longer follow-ups is necessary. Compared with other anti-incontinence surgery, the mid-urethral sling procedure may have the least complication rate and risks of postoperative voiding dysfunction. Conservative methods can successfully manage most scenarios of postoperative voiding dysfunction after the mid-urethral sling procedure, including medications, intermittent catheterization, timed toileting, pelvic muscle exercises, or Hegar dilation. Once the symptomatic urethral obstruction is confirmed, the release of the mid-urethral tape is necessary. At six to thirty-six months of the tested group, the SUI cure rates were 87.1% and 88.9%, respectively. Both groups’ approximate SUI cure rates convinced us of the efficacy of the Long-loop in resolving iatrogenic urethral obstruction without sacrificing the urinary continence resulting from the mid-urethral sling.

The Long-loop manipulation procedure provided an alternative to cutting the mesh for releasing obstruction. The cutting extent could be challenging to manage and may create wounds and pain for the patient. Cutting not enough might lead to repeating the procedure, while cutting too much, failing to remain continent, might make patients unsatisfied and untrusting. Midline or lateral excision of the mid-urethral sling requires local anesthesia and vaginal incision, which may expose the patients to bleeding or infection risks. On the contrary, the Long-loop manipulation procedures avoid creating new wounds, and no anesthesia is necessary. Worth noting that the Long-loop manipulation procedure could still be performed a week after the operation of SUI, which gives us more time to monitor the patient’s clinical condition and adjust if necessary. The Long-loop manipulation procedure is a cost-down compared to all the other resolutions when an iatrogenic voiding dysfunction occurs.

For patients choosing a single-incision sling system, it is not only the effects on treating SUI that they persuade but also the reduction of postoperative wound pain and discomfort. Hence, it could be problematic for these patients to receive invasive surgical treatment for such postoperative complications. Therefore, the tape-releasing suture may play a role as a backup plan.

One patient in our study failed to remain continent after the first Long-loop manipulation procedure. In contrast, two patients who received more than one Long-loop manipulation procedure succeeded, suggesting that the Long-loop manipulation procedure should be conducted subtly. Other than over-tension resulting from the surgeon’s judgment or experience, multiple factors should also be considered that could cause iatrogenic urethral obstruction, such as anesthesia, edema, and pain, which could gradually and spontaneously resolve a few days later [26]. Recently, the popularity of non-deformable mid-urethral slings such as I-STOP has increased. According to the mechanism of resolving the over-tension from the Long-loop, which is utilizing traction force to loosen the sling, the efficacy of the Long-loop on non-deformable slings seems limited mechanically and may require more studies.

From the questionnaires we employed, both groups, either with or without the Long-loop manipulation procedure, showed significant improvement at six months follow-up (Table 3). Though the OAB symptom score (OABSS) had been improved significantly in both groups, OAB itself did not show significant differences within the group with Long-loop manipulation procedures, which can be considered a statistical bias because of the small number of patients in the group. A similar result is revealed in Table 4. As detrusor overactivity (DO) had a notable change after the operation in the group without the Long-loop manipulation procedure, there was no specific change in the group with the Long-loop manipulation procedure that only had 2 participants. The first sensation to void checked in our routine urodynamic exams found significant improvement six months after the operation in the group without the Long-loop manipulation procedure. The group with the Long-loop manipulation procedure showed a marginal improvement (*p* = 0.07). Though current studies and literature show abundant evidence of improving OAB after SUI surgery; however, the exact mechanism remains unclear [27,28].

As for the impact of the Long-loop manipulation procedure on vaginal irritation, we had received no complaints since most patients had it removed within two days post-operation. Also, in our suture technique, we left two single sutures below the Long-loop and one single suture above it over the vaginal epithelium, which left the Long-loop to stand out more from the vaginal introitus. Only one patient had her third release one-week post-operation, but no apparent vaginal erosion or irritation was mentioned. During this study’s follow-up period, no sling exposure was observed or complained so far. The traction downward of the Long-loop is subtle and slightly loosens the sling’s tension, which did not pull the sling out from the vaginal wound. Hence, sling exposure could be possible but less likely; a longer follow-up is necessary.

The strengths of our study are standard evaluation protocol with adopting validated questionnaires and objective urodynamic evaluation. The limitation of our study is a relatively large sample size (a total of 149 patients) but with only nine patients (6%) that had suffered from iatrogenic urethral obstruction. However, urethral obstruction after the mid-urethral surgery is relatively uncommon, as reported in the current literature [14,21]. Whether a cut-off point for diagnosing urethral obstruction post-operatively remains controversial since postoperative edema, inflammation, and anesthesia or concomitant surgery are also risk factors of iatrogenic urethral obstruction and might affect the natural urination process shortly. A well-accepted cut-off point for the diagnosis of urethral obstruction requires further investigation.

The success of the mid-urethral sling largely depends on the operators’ experience; meanwhile, the Long-loop manipulation procedure requires the same as well. The traction force from the Long-loop manipulation procedure is operator-dependent, and only the operator from the SUI surgery should know the intensity of the mid-urethral sling that he/she provided. Though the simplicity and efficacy seem better than other surgical treatments for iatrogenic urethral obstruction, the action of traction of the tape-releasing sutures requires clinical experience and skills as the other procedures similarly. Also, identifying the risk factors for iatrogenic urethral obstruction is essential.

## 5. Conclusions

We are convinced of the practicability and efficacy of the tape-releasing suture “Long-loop”. We adopted subjective and objective means to evaluate both groups before and after a six-month follow-up. The Long-loop manipulation procedure can successfully resolve the iatrogenic urethral obstruction without compromising the effectiveness of mid-urethral sling for the treatment of SUI. The Long-loop manipulation procedure can be performed multiple times post-operatively and one week after the surgery, just before the wound healing and fibrosis formation. Nonetheless, whether the Long-loop can be applied on a non-deformable sling should undergo further investigation.

## Figures and Tables

**Table 1:** Clinical background of women with stress urinary incontinence in both groups. Data are given as mean ± standard deviation, or *n* (%).

	T (*n* = 140)	TR (*n* = 9)	*p* Values
Mean age (years)	59.6 ± 11.5	64.6 ± 8.3	0.21 *
Mean parity	2.8 ± 1.2	3.1 ± 0.9	0.49 *
Mean BMI (kg/m^2^)	25.3 ± 3.6	25.0 ± 4.4	0.79 *
Menopause	112 (80)	9 (100)	0.21 **
Current hormone therapy	7 (5)	0 (0)	1.0 ^
Diabetes Mellitus	28 (20)	4 (44.4)	0.1 ^
Hypertension	55 (39.3)	3 (33.3)	1.0 ^
History of hysterectomy	33 (23.6)	0 (0)	0.21 ^
Intrinsic sphincter deficiency	22 (15.7)	1 (11.1)	0.71 *
Pre-op pad test	32.6 ± 20.4	41.2 ± 25.6	0.1 *
Mid-urethral slings TVT-O	100 (71.4)	7 (77.8)	0.68 **
Solyx	29 (20.7)	2 (22.2)	0.91 ^
Ophira	11 (7.9)	0 (0)	0.38 ^
Concomitant procedures			
Anterior repair	23 (16.4)	2 (22.2)	0.65 ^
Anterior repair + TVM surgery	30 (21.4)	3 (33.3)	0.41 ^
Vaginal hysterectomy	28 (20.0)	1 (11.1)	0.51 ^
Laparoscopic hysterectomy	13 (9.3)	0 (0)	1.0 ^
Follow-up (months)	24.8 ± 6.3	21.4 ± 5.6	0.38 *

T, tape; TR, tape-releasing; Pre-op, preoperative; TVM, transvaginal mesh; * Student’s *t*-test; ^ Fisher’s exact test; ** Chi-square test.

**Table 2:** Surgical Results by Symptomatology.

Symptom or Sign	Pre-op	Post-op	After Release
Frequency or urgency	4	8	1
Straining to void	0	9	0
Postvoid residual urine > 100 mL	1	9	0
Stress urinary incontinence	9	0	1

**Table 3:** Urinary symptoms of patients with stress urinary incontinence in both groups before and 6 months after surgery. Data are given as *n* (%).

	T (*n* = 140)			TR (*n* = 9)		
	Pre-op	Post-op	*p* Value	Pre-op	Post-op	*p* Value
OAB	59 (42.1)	27 (19.3)	<0.001 *	4 (44.4)	2 (22.2)	0.17 *
OABSS	5.7 ± 2.5	2.2 ± 1.6	<0.01 *	5.5 ± 3.1	3.0 ± 1.2	0.026 **
UDI-6	26.2 ± 14.2	6.9 ± 3.6	<0.001 *	32.6 ± 16.4	15.8 ± 7.0	<0.01 **
IIQ-7	18.1 ± 10.4	3.2 ± 2.6	<0.001 *	22.5 ± 10.4	8.1 ± 6.6	0.004 **
ICIQ-SF	9.6 ± 5.6	3.1 ± 1.3	<0.001 *	9.0 ± 3.2	4.5 ± 3.3	<0.01 **
Pad test (g)	29.3 ± 8.5	0.3 ± 0.2	<0.001 *	41.1 ± 27.0	0.4 ± 0.2	<0.01 **

SUI, stress urinary incontinence; UUI, urgency urinary incontinence; UDI-6, Urogenital Distress Inventory; IIQ-7, Incontinence Impact Questionnaire. * McNemar’s test, ** Paired *t*-test.

**Table 4:** Urodynamic changes in both groups before and 6 months after surgery. Data are given as *n* (%) or mean ± standard deviation.

	T (*n* = 140)			TR (*n* = 9)		
	Pre-op	Post-op	*p* Value	Pre-op	Post-op	*p* Value
DO	31 (22.1)	6 (4.3)	<0.01 *^	2 (22.2)	0 (0)	0.16 *
Qmax (mL/s)	24.2 ± 10.0	22.4 ± 8.6	0.78 **	23.0 ± 11.3	22.6 ± 8.9	0.66 **
RU (mL)	21.2 ± 12.3	28.8 ± 11.7	0.28 **	26.3 ± 10.0	27.6 ± 15.6	0.38 **
FS (mL)	133.5 ± 36.9	175.9 ± 87.2	<0.01 **^	159.5 ± 40.4	168.6 ± 32.4	0.07 **
MCC (mL)	290.4 ± 53.2	303.7 ± 93.2	0.25 **	312.4 ± 65.7	308.2 ± 61.6	0.22 **
Pdet (cmH_2_O)	18.8 ± 10.3	20.6 ± 8.7	0.41 **	19.5 ± 7.8	18.3 ± 10.4	0.61 **
FUL (mm)	26.2 ± 9.0	28.3 ± 7.5	0.37 **	28.4 ± 8.3	27.4 ± 9.4	0.19 **
MUCP (cmH_2_O)	67.1 ± 22.4	65.7 ± 23.0	0.28 **	58.6 ± 24.5	56.9 ± 21.7	0.46 **
UCA (mmcmH_2_O)	1160.0 ± 309.2	1207.4 ± 398.3	0.32 **	1080.6 ± 490.8	996.1 ± 392.4	0.36 **

DO, detrusor overactivity; Qmax, maximum flow rate; RU, residual urine; FS, first sensation to void; MCC, maximum cystometric capacity; Pdet, detrusor Pressure at peak flow; FUL, functional urethral length; MUCP, maximum urethral closure pressure; UCA, urethral closure area. * McNemar’s test; ** Paired *t*-test. ^ Statistical significance.

## Data Availability

The data presented in this study are available on request from the corresponding authors. The data are not publicly available due to privacy.
